# Sympathetic overactivity and nocturnal diuresis in obstructive sleep apnea alter the response to hypertension therapy

**DOI:** 10.1186/s40885-024-00272-x

**Published:** 2024-06-01

**Authors:** Michael G. Ziegler, Milos Milic, Joel E. Dimsdale, Paul J. Mills

**Affiliations:** 1 Department of Medicine, Division of Nephrology and Hypertension, University of California San Diego, UCSD Medical Center, 200 W. Arbor Drive, San Diego, CA 92103- 8341 USA; 2 Department of Psychiatry, University of California San Diego, UCSD Medical Center, 200 W. Arbor Drive, San Diego, CA 92103-8341 USA; 3 Department of Family Medicine and Public Health, University of California San Diego, UCSD Medical Center, 200 W. Arbor Drive, San Diego, CA 92103-8341 USA

**Keywords:** Norepinephrine, Flow mediated dilation, Guanfacine, Hydrochlorothiazide, Baroreflex, Ambulatory blood pressure, Heart rate variability

## Abstract

**Background:**

Obstructive sleep apnea (OSA) is associated with high blood pressure that responds poorly to usual antihypertensive therapy.

**Methods and results:**

Forty-one subjects with OSA had 25% higher plasma norepinephrine and 42% higher epinephrine measured every 2 h over 24 h than 20 control subjects. They also excreted more sodium during sleep. This suggested that that a sympatholytic would be a more successful antihypertensive than a diuretic. To test this hypothesis we treated a second group of 23 hypertensive apneics with placebo, 6 weeks of the sympatholytic guanfacine and 6 weeks of hydrochlorothiazide in a crossover study. Guanfacine lowered 24-hour blood pressure by 9.6/6.7 mmHg, more than the 5.4/2.9 mmHg effect of hydrochlorothiazide (*P* < 0.05). Nighttime systolic blood pressure dipping was poor at 6.6 ± 1.8%. Hydrochlorothiazide did not alter blood pressure dipping but guanfacine improved dipping to 9.1 ± 1.2%, a better result (*P* = 0.03) than from the diuretic. Central aortic pressure by pulse wave analysis was 120/84 mmHg on hydrochlorothiazide and 109/72 on guanfacine, (*P* < 0.05). Guanfacine, but not hydrochlorothiazide, improved baroreflex sensitivity, heart rate variability and flow mediated vascular dilation, suggesting that decreasing the elevated sympathetic nerve activity of obstructive sleep apnea returned vascular function toward normal.

**Conclusions:**

OSA is the most common condition associated with antihypertensive treatment failure. It increased sympathetic nerve activity day and night. Drugs that block sympathetic nerve function are not among the 4 most commonly recommended classes of antihypertensives but diuretics are. Sympatholytic therapy was superior to diuretic treatment for hypertension associated with sleep apnea.

**Trial registration:**

NCT, NCT02699125, Registered 26 February 2016 - Retrospectively registered, https://clinicaltrials.gov/study/NCT02699125.

**Supplementary Information:**

The online version contains supplementary material available at 10.1186/s40885-024-00272-x.

## Introduction

Obstructive sleep apnea (OSA) is strongly associated with hypertension [1]. Fifty to 90% of patients with treatment resistant hypertension have OSA, making it by far the most common condition associated with treatment failure [2]. The reason for the resistance to antihypertensive therapy is unknown. Apneas are often followed by brief spikes in blood pressure (BP) but the source of sustained BP elevation throughout the day is less clear. Apneic episodes trigger a spike in sympathetic nerve electrical activity and BP and there is a nocturnal increase in plasma and urine norepinephrine and the urinary norepinephrine metabolite normetanephrine [3, 4]. Blockade of β1-adrenergic receptors in hypertensive subjects with OSA lowered 24-hour BP moderately [5]. Svedmyer et al. [6] found in a retrospective analysis of the European Sleep Apnea Database that treatment with a beta blocker was associated with the lowest systolic BP. However, current hypertension treatment guidelines relegate sympatholytic drugs to fourth or fifth line therapy [7]. Patients with OSA complain of nocturia and hypertensive patients with OSA had a nocturnal natriuresis [8]. Apneas decrease intrathoracic pressure, increasing venous return to the heart. Some [9], but not all [10–12] investigators found increased nocturnal atrial natriuretic peptide (ANP) and B-type natriuretic peptide (BNP) levels, which might be expected from nocturnal cardiac dilation. The elevated BP associated with apneas might also elicit a nocturnal pressure diuresis [9]. We first studied subjects with OSA to better define whether those with high BP have only a nocturnal or a sustained increase in sympathetic neurotransmitters and if they have a nocturnal natriuresis (Study 1). Based on the positive results of that study, we designed a second study (Study 2) to see if a sympatholytic could control BP in OSA better than a diuretic. We also hypothesized that a treatment that returned sympathetic nerve activity toward normal would also normalize functional responses of the arteries, so we measured flow mediated dilation, pulse wave velocity and physiologic responses to the baroreflex.

## Materials and methods

Study 1. Forty-one women and men with untreated OSA and 20 healthy adults participated in study 1 (Table S1). Subjects were excluded if they reported a history of a condition (other than OSA and hypertension) that might interfere with their ability to complete the study, that would interfere with data collection or analysis or that might make it unsafe to discontinue medications. Subjects with cardiac arrhythmia, heart failure, diabetes, renal insufficiency, hepatic or endocrine disorders or secondary causes of hypertension were not eligible. Subjects in Study 1 all had an apnea-hypopnea index > 10 on polysomnography carried out in the Clinical Research Center. Patients who were receiving anti-hypertensive medications had their medications tapered for 3 weeks prior to participation. Study 1 and study 2 (below) were approved by the University of California San Diego (UCSD) Human Subjects Committee. After description of the study, written informed consent was obtained. We scored obstructive apneas as previously described [13, 14]. The participants arrived at the UCSD Clinical Research Center at 5:00 p.m., at which time a venous catheter was inserted. Starting at 6:00 p.m. a blood sample was collected every 2 h for the next 24 h, spun in a refrigerated centrifuge, and the plasma was stored at − 80 °C. Subjects provided urine over four six hour samples. The urine sodium was calculated per gram creatinine to diminish errors due to incomplete voiding in older males as previously described [15]. We measured plasma norepinephrine (NE) and epinephrine (E) by the technique of Kennedy and Ziegler [16]. Study 2 was a blinded crossover study comparing the antihypertensive efficacy of HCTZ and the α2-adrenergic agonist guanfacine in adult subjects with a diagnosis of moderate to severe OSA with both an apnea-hypopnea index (AHI) > 10 and hypertension. Hypertension was defined as a systolic blood pressure (SBP) ≥ 140 mmHg and/or a diastolic blood pressure (DBP) ≥ 90 mmHg on the average of three seated BP measurements taken in a quiet room in a research setting after the subject had time to accommodate to surroundings, or a history of pharmacologic therapy for hypertension. Subjects in Study 1 had an AHI of 39 ± 30 and oxygen saturation below 90% at 7.1% of sleep time. Study 2 subjects were representative of patients with OSA with a diagnosis of at least moderate OSA or an AHI > 10 (Table S3). Subjects with SBP/DBP ≥ 180/105 mmHg at screening or during medication tapering were excluded for safety. Patients were excluded if they needed medications known to influence the autonomic nervous system, heart rate or sleep medicines. No subjects worked nights. Antihypertensives were discontinued with home or clinic BP monitoring for two weeks prior to study. Then, a single blind placebo was taken for two weeks followed by six weeks of either guanfacine or hydrochlorothiazide (HCTZ) in random order. Then, medications were crossed over and taken for another six weeks. Each medication was taken as one capsule (either guanfacine 0.5 mg/day or HCTZ 12.5 mg/day) for two weeks and then two capsules (either guanfacine 1 mg/day or HCTZ 25 mg/day) for the remaining four weeks. All medications were taken at night. Medication compliance was evaluated by pill count. We used the Epworth Sleepiness Scale (ESS) for a subjective assessment of daytime sleepiness [17].

### Blood pressure measurements

We measured screening BP according to AHA recommendations [18]. Following placebo and active drug treatment subjects wore an oscillometric ambulatory blood pressure monitor (ABPM) for 24-h (SpaceLabs model 90,207) programmed to measure BP every 15 min during the wake period and every 30 min during the sleep period. The 24 h ambulatory blood pressure monitoring (ABPM) recordings were accepted for analysis if they provided more than 70% successful readings. We used Spacelabs Medical 92,506 ABP RMS Software (V3.0.0.9, Spacelabs Medical, Redmond, WA, USA) and custom made LabVIEW software (National Instruments, Austin, TX) for data analysis. Subjects kept a diary to define wake and sleep periods. On the day of ABPM, the blood pressure cuff was applied on the nondominant arm between noon and 1 p.m. and removed the following day between noon and 1 p.m. We instructed subjects to keep their daily routine except to refrain from exercise, and to keep their cuffed arm immobile during each recording [19].

### Pulse wave velocity and pulse wave analysis

We determined arterial stiffness by measuring carotid-femoral pulse wave velocity (cfPWV) with a Millar applanation tonometer attached to the SphygmoCor system (AtCor Medical, Sydney, Australia). PWV was determined from the ECG R-wave gated right carotid artery pulse and right femoral pulse, corresponding transit times and the distance between the two arterial sites. We determined PWV for each subject as an average of three consecutive measurements in supine position. Central pulse wave analysis (PWA) was performed in the sitting position from the right radial artery with a Millar applanation tonometer and the SphygmoCor system [20].

### Spontaneous baroreflex sensitivity

Subjects had beat-to-beat BP and heart rate (HR) recorded from their right middle finger while supine for 5 min by using finger photoplethysmography with Finometer (FMS, Finapres Measurement Systems, Amsterdam, The Netherlands). We used the sequence method and custom made LabVIEW software to calculate spontaneous cardiovagal baroreflex sensitivity (cBRS) [21, 22]. Briefly, we calculated the slopes of the linear regression lines between the SBP and the subsequent interbeat intervals for all valid sequences where SBP and interbeat interval values either increased or decreased within 3 or more consecutive beats. Valid sequences had at least a 1 mmHg SBP change and 5 ms interbeat interval change between beats and a correlation coefficient *R* ≥ 0.85. Finally, we calculated the overall spontaneous baroreflex sensitivity as an average of all valid baroreflex sequences.

### Heart rate variability

We used the SphygmoCor (V7.1, AtCor Medical, Sydney, Australia) to record ECG and HR for five minutes in resting supine position. Then we used SphygmoCor built-in software to analyze the recorded ECG signal and calculate short term (5 min) heart rate variability (HRV). We quantified HRV in the time domain by the root mean square of successive RR interval differences (RMSSD). In the frequency domain we analyzed HRV in two frequency bands, the low frequency (LF) band (0.04 –0.15 Hz) and in high frequency (HF) band (0.15 –0.4 Hz) to determine LF power, HF power and total power as well as sympathovagal balance (LF/HF ratio) [23].

### Flow-mediated dilation

After 15 min of relaxation in the supine position, an occlusion cuff was placed on the right forearm and the brachial artery was scanned in longitudinal Sects. 4–10 cm proximal to the antecubital fossa by using a 5–12 MHz linear-array ultrasound probe (HDI 5000 Ultrasound System, Bothell WA) mounted on a custom made probe holder. ECG R-wave triggered beat-to-beat duplex ultrasound images of blood flow velocity and brachial artery diameter were acquired and stored with IMAQ Vision Builder (V 6.1, National Instruments, Austin, TX) during one minute pre-occlusion baseline period and during three minutes post-occlusion reactive hyperemia. Brachial artery diameter and linear blood velocity were analyzed beat-to-beat with semi-automated Brachial Analyzer for Research software (Medical Imaging Applications, LLC Coralville, IA). Flow mediated dilation (FMD) was calculated as

$$FMD= \left(\frac{{D}_{max}}{{D}_{baseline}}\right)-1$$

where *D*_*max*_ (cm) is maximal post occlusion diameter and *D*_baseline_ (cm) is average pre-occlusion baseline diameter. Beat-to-beat pre and post occlusion shear rate *γ* (s^− 1^) was calculated as

$$\gamma =4 \text{x} \frac{{V}_{avg}}{D}$$

where *V*_*avg*_ (cm/s) is average blood velocity during each cardiac cycle and *D* (cm) is the corresponding arterial diameter. Normalized FMD (nFMD) was calculated by dividing FMD with cumulative shear rate, expressed as the AUC, up to the point of maximal dilation [24].

$${FMD}_{normalized}=\frac{FMD}{AUC}$$

### Statistical analysis

Results were examined by one-way repeated measures ANOVA. A significant main effect of treatment within the ANOVA was further assessed with a Tukey post hoc test. Non-normally distributed values were analyzed by ANOVA on ranks. Statistical analysis was completed by using SigmaPlot 11 (Systat Software, Chicago, IL, USA). Data are presented as means ± sem unless stated otherwise. Study 2 was registered with ClinicalTrials.gov NCT02699125 with 24-h ABP as the primary outcome measure and nFMD as secondary outcome measure.

## Results

### Study 1

**Fig. 1:**
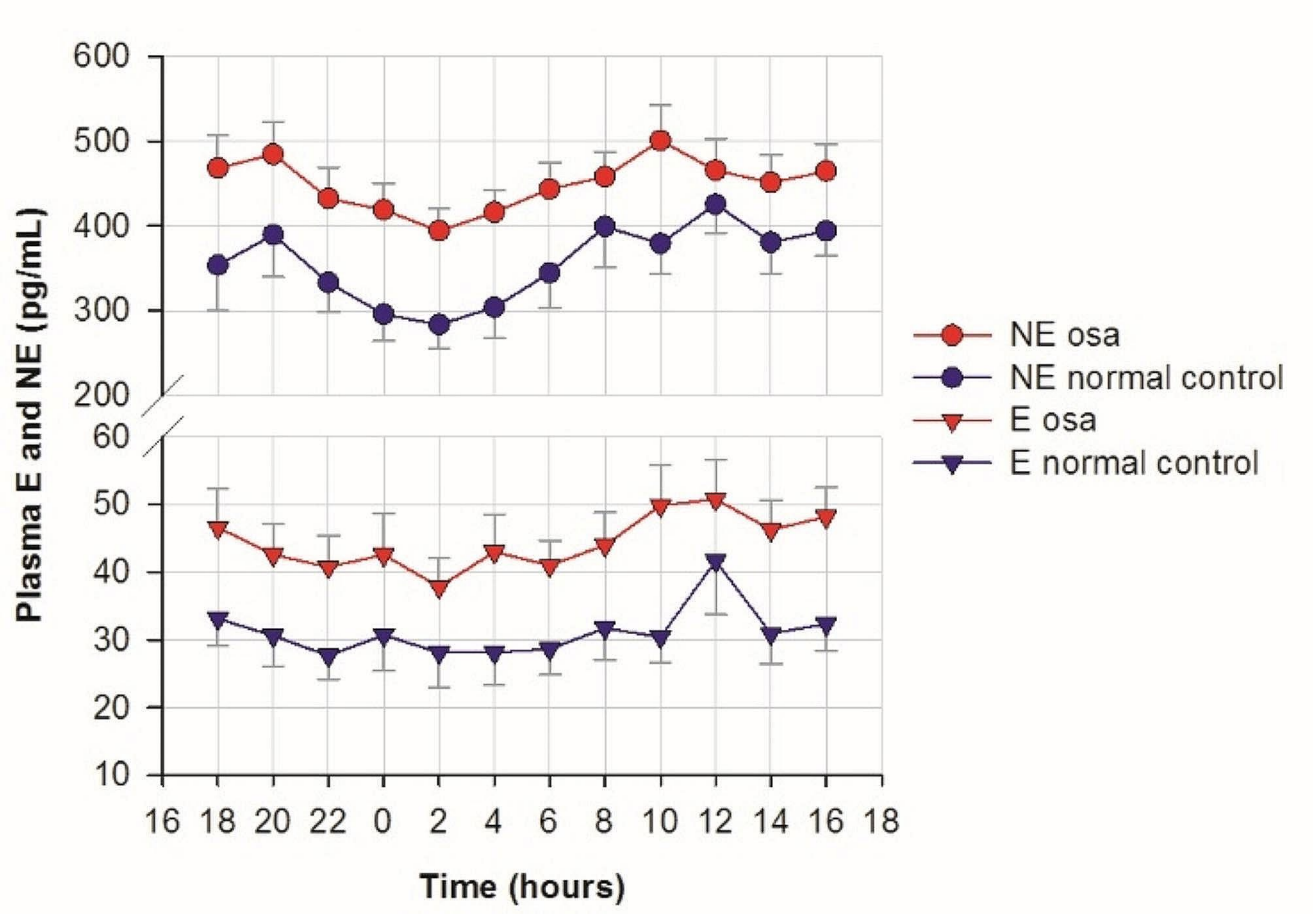
24-h plasma norepinephrine (NE) and epinephrine (E) concentration in normal controls and OSA (mean ± sem). The concentration of NE and E in plasma was analyzed by sampling blood every two hours for 24-hours in the Clinical Research Center. All participants, 20 controls and 41 apneics, had all 12 NE and E data points. The shaded area indicates the sleep period. Both NE and E were higher in OSA than in normal controls (*P* < 0.001)

Subjects with OSA in study 1 were slightly younger than control subjects, but slightly more overweight and had a higher SBP. Their mean AHI of 39 ± 30 is in the severe OSA range. Control subjects mean AHI of 5.4 ± 2.8 resides at the borderline between normal and mild OSA (Table S1). Subjects with OSA in study 1 had a higher urine sodium to creatinine ratio during sleep than control subjects (Figure S1). Average 24-h plasma NE was 25% higher in OSA than in control subjects. It was 33% higher during sleep and 21% higher while awake. Average 24-h plasma E was 42% greater in OSA (Fig. 1 and Table S2).

### Study 2

The 23 subjects in study 2 all had diagnoses of OSA and hypertension. They were predominantly male and overweight (Table S3) both characteristic of subjects with OSA.

### 24-h ambulatory blood pressure monitoring (ABPM)

Treatment of these subjects with guanfacine lowered BP by 9.6/6.7 mmHg, more than the 5.4/2.9 mmHg effect of HCTZ both while awake (*P* < 0.05) and asleep (*P* < 0.05) for both SBP and DBP (Fig. 1; Table 1 and Table S4). Guanfacine lowered overall 24-h HR (-4.9 ± 0.8 bpm) while HCTZ did not affect HR, (*P* < 0.05, Table 1). Neither drug altered BP variability (BPV) measured as wake or sleep SD (Table S5). The overall 24-h diastolic BPV, calculated as the weighted SD [25] with fixed duration of the daytime SD (14 h) and nighttime SD (6 h) (wSDf), increased after guanfacine by 12% (from 7.17 mmHg placebo to 8.03 mmHg guanfacine, *P* = 0.024). The 24-h diastolic BPV weighted SD, calculated with variable duration of sleep and wake periods (wSDv) increased by 9% (from 7.03 mmHg placebo to 7.95 mmHg guanfacine, *P* = 0.025) (Table S6).

**Table 1:** Change from placebo in BP and HR during the overall 24-h period, wake period and sleep period in response to HCTZ or guanfacine. BP values are plotted in Fig. 2

	Treatment			*P*-value
Variable	Placebo	Δ Guanfacine	Δ HCTZ	Δ Guanfacine vs. Δ HCTZ
Overall 24 h				
SBP (mmHg)	143 ± 0.95	-9.6 ± 0.8	-5.4 ± 0.7	< 0.05
DBP (mmHg)	87 ± 1.08	-6.7 ± 0.5	-2.9 ± 0.5	< 0.05
HR (bpm)	81 ± 1.6	-4.9 ± 0.8	0.6 ± 0.8	< 0.05
Wake period				
SBP (mmHg)	146 ± 1.06	-10.2 ± 0.5	-6.9 ± 1.4	< 0.05*
DBP (mmHg)	91 ± 1.53	-7.3 ± 0.6	-3.7 ± 1	< 0.05
HR (bpm)	87 ± 1.04	-2.7 ± 0.7	-2.7 ± 0.	< 0.05
Sleep period				
SBP (mmHg)	136 ± 0.69	-9.8 ± 1.7	-5 ± 1.1	< 0.05
DBP (mmHg)	82 ± 1.32	-6.8 ± 1.4	-3.1 ± 0.7	< 0.05*
HR (bpm)	72 ± 0.84	-2.6 ± 0.9	2.7 ± 0.7	< 0.05

Δ: change from placebo value, *: Friedman RM ANOVA on Ranks

### Blood pressure dipping

While taking placebo, SBP dipping was poor at 6.6 ± 1.8%. Dipping remained the same during HCTZ treatment at 6.6 ± 1.4%. Dipping improved with guanfacine to 9.1 ± 1.2%, better than with HCTZ (*P* = 0.03) but still less than the 10–20% considered optimal (Table S7).

### Central hemodynamics

Following guanfacine, aortic SBP decreased by 9% (from 120 mmHg on placebo to 109 mmHg on guanfacine, *P* < 0.05). Central aortic workload (normalized augmentation index, aAIx @ HR75) fell from 19% placebo to 14.7% on guanfacine, *P* < 0.05. Guanfacine improved cardiac contractility measured as fractional ejection duration (ED%) by 3% (from 35% placebo to 32% guanfacine, *P* < 0.05). Finally, subendocardial oxygen supply/demand ratio improved from 161% placebo to 180% guanfacine (Table S8). The carotid-femoral pulse wave velocity (cfPWV) was 7.6 ± 0.4 m/sec on placebo, 7 ± 0.3 m/s on guanfacine and 7.3 ± 0.4 m/s on HCTZ (*P* = NS, Table S9).

### Heart rate variability (HRV)

Guanfacine increased HRV in time domain (RMSSD) from 36.7 ms after placebo to 42.9 ms after guanfacine *P* < 0.05. No significant effect was found by use of frequency domain HRV indexes (Table S10).

### Spontaneous cardiovagal baroreflex sensitivity

Spontaneous cardiovagal baroreflex sensitivity (cBRS) while taking placebo was 7.7 ± 0.9 ms/mmHg. It increased to 11.4 ± 1.6 ms/mmHg while subjects took guanfacine (*P* < 0.05) and was only 8.5 ± 1.6 ms/mmHg while taking HCTZ (*P* = NS) (Table S11).

### Flow mediated dilation (FMD)

Baseline brachial artery diameter (Db) did not change throughout the study. The maximal increase in diameter during the post-occlusion reactive hyperemia (ΔD) increased on guanfacine from 0.23 mm after placebo to 0.36 mm after guanfacine, *P* < 0.05, but not after HCTZ. Shear rate normalized FMD (nFMD) increased by 3.9% on guanfacine from 4.5% after placebo to 8.4% after guanfacine, (*P* < 0.05, Table 2).

**Table 2:** Effect of treatment on brachial artery flow mediated dilation (FMD).

Treatment				*P*-value		
Variable	Placebo	Guanfacine	HCTZ	Guanfacine vs. Placebo	HCTZ vs. Placebo	HCTZ vs. Guanfacine
Db (mm)	4.19 ± 0.19	4.18 ± 0.17	4.18 ± 0.17	NS	NS	NS
ΔD (mm)	0.23 ± 0.02	0.36 ± 0.04	0.28 ± 0.03	< 0.05	NS	NS
nFMD (%)	4.5 ± 0.8	8.4 ± 1.1	4.9 ± 0.7	< 0.05	NS	NS

Db: baseline diameter, ΔD: maximum diameter change, nFMD: shear rate normalized FMD.

### Sleepiness

We used the Epworth Sleepiness Scale (ESS) for a subjective assessment of daytime sleepiness [17]. An ESS score > 10 indicates the presence of sleepiness. The ESS was 10.2 ± 1.1 on placebo, 9.6 ± 1.1 on guanfacine and 10.5 ± 1 on HCTZ (*P* = NS).

## Discussion

Subjects with OSA in Study 1 had increased plasma NE and E all day and night along with a nocturnal natriuresis. This suggested that a sympatholytic would be an effective antihypertensive. Since subjects with OSA have a nocturnal diuresis we expected that adding a pharmacologic diuretic would be less effective at lowering BP than a sympatholytic. In Study 2 subjects with OSA and hypertension lowered 24-hour BP and improved BP dipping more in response to the sympatholytic guanfacine than the diuretic HCTZ.

Subjects with OSA in study 1 were slightly heavier and had higher BP than control subjects, as is characteristic of those with OSA (Table S1). OSA causes nocturnal spikes in sympathetic nerve activity and urine NE [3, 14]. We find that plasma NE and E are elevated throughout both day and night in OSA subjects. While volume contraction from a nocturnal natriuresis might increase NE, it would not be expected to increase E, which was even more elevated. Animal studies show that short stressors lead to prolonged increases in NE and E synthesizing enzymes [26]. Some human stressors are also associated with prolonged increases in NE and BP [5]. The stress of nocturnal apneas seem a likely explanation for the 24-hour increase in NE and E in OSA Subjects with OSA have frequent daytime drowsiness and this can make them appear relaxed. However, we find that they have increased daytime neuronal NE levels and adrenal E levels, indicating an activated sympathetic nervous system.

The hypertension of OSA often responds poorly to pharmacotherapy [2]. To test whether our subjects with both OSA and hypertension would respond better to a sympatholytic than to a diuretic we selected treatment with a standard 25 mg dose of the commonly used diuretic HCTZ and compared that with a low 1 mg dose of guanfacine, which is supplied in 1–4 mg dosage forms. Guanfacine stimulates central α2-adrenergic receptors inhibiting sympathetic nerve activity and lowering plasma NE. Its 17 h half-life permits once nightly dosing providing slightly higher nighttime blood drug levels and improved BP dipping.

**Fig. 2:**
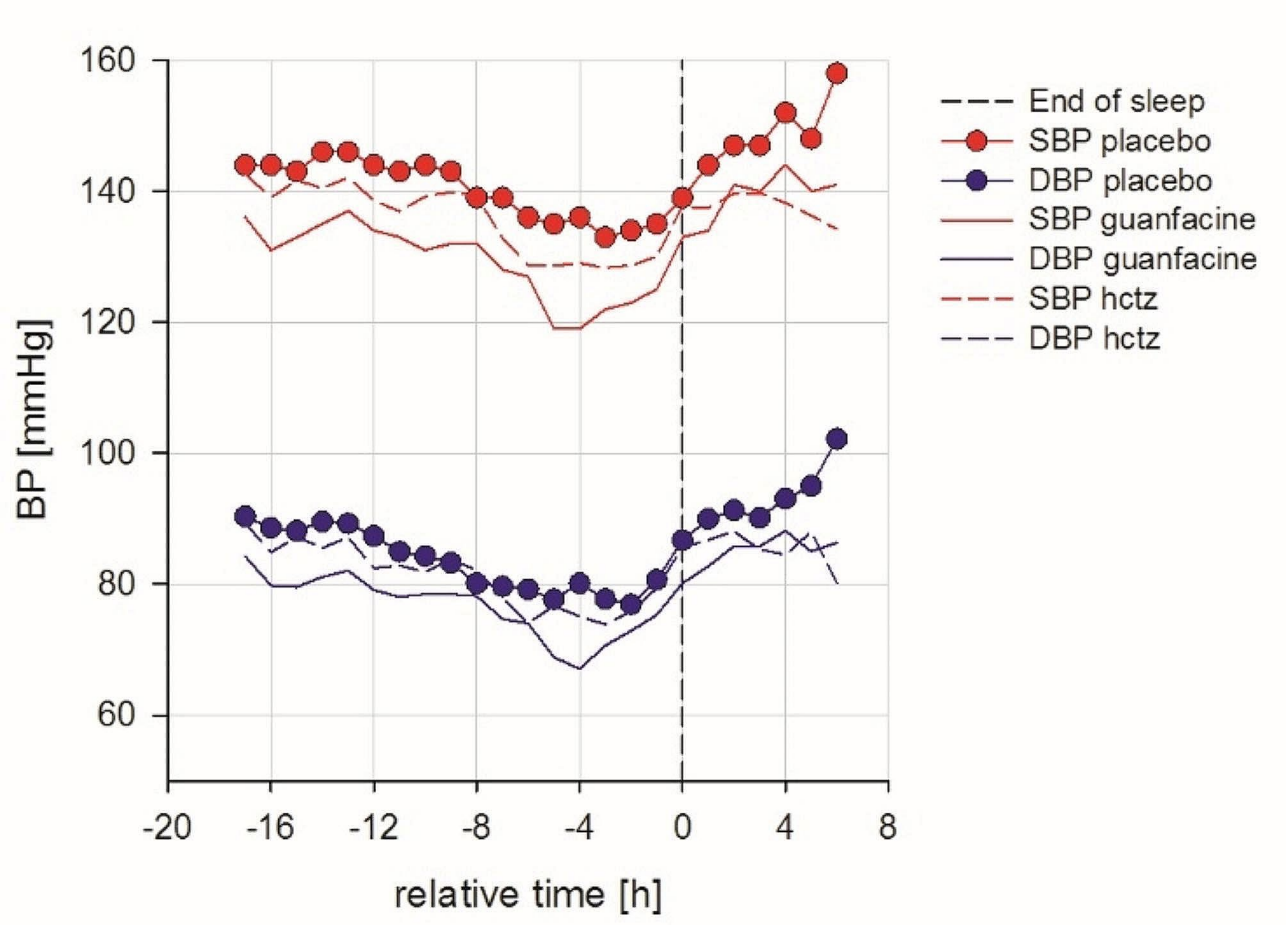
24-h systolic SBP and DBP adjusted to wake time for placebo, guanfacine and HCTZ. All data are centered at the end of sleep time (wake time = 0 h on the X-axis). Since arbitrary nighttime and daytime definitions can cause dipping status misclassification, we used a sleep diary to determine actual sleep and awake hours [38]. Guanfacine lowered SBP and DBP more than HCTZ during the wake period, sleep period and over 24-hours (*P* < 0.05)

Guanfacine lowered BP by 9.6/6.7 mmHg; HCTZ lowered BP by 5.4/2.9 mmHg. The mean BP decrease from a single drug in patients with BP similar to our study was about 7/5 mmHg [27]. A retrospective study of the European Sleep Apnea Database [6] reported that subjects with OSA have a better BP lowering response to beta blockers than with other drugs, including drugs that act like guanfacine, and to diuretics. Our prospective study is in accord with reports of the efficacy of beta blockers but adds that guanfacine was superior to HCTZ. The subjects who received HCTZ and guanfacine had a mean age of 52 and only one was African American. Young white hypertensives tend to be less sensitive to the hypotensive effects of diuretics, and that was seen in our subjects. The study size and age of subjects did not provide sufficient statistical power to determine if HCTZ or guanfacine BP lowering effect was altered by either age or by sodium intake. Subjects with OSA have increased sympathetic nerve tone, both beta blockers and guanfacine decrease the effects of that nerve activity. Subjects with OSA have diminished BP dipping [28]. Guanfacine treatment led to SBP dipping of 9.1%, better than HCTZ. Nocturnal BP and BP dipping are independent risk factors for cardiovascular disease [29]. The effect of HCTZ on nocturnal BP was modest but guanfacine had a better nighttime effect (Fig. 2; Table S4) that may be attributed to inhibition of the nocturnal spikes in sympathetic nerve activity that accompany apneas. Our study and others suggest that only some antihypertensives will improve dipping, even when taken at night [29, 30]. Addition of a diuretic to the nocturnal natriuresis of subjects with OSA seemed unlikely to improve dipping. On the other hand, sympatholytic therapy seemed especially suited to treatment of the pressor effect of apneas. There are several classes of sympatholytic antihypertensive in common use; α-blockers, β-blockers, α-β blockers and α_2_-agonists such as guanfacine. Treatment resistant hypertensives have evidence of heightened sympathetic activity as a cause of treatment failure [31]. Treatment resistant hypertensives often have OSA but sympatholytics are often not among recommended agents when BP fails to respond to treatment [2, 32].

Increased sympathetic activation in patients with OSA is associated with decreased baroreflex sensitivity, impaired endothelial function, and decreased heart rate variability. Guanfacine therapy returned these three toward normal. Guanfacine increased resting supine cardiovagal baroreflex sensitivity by 47.7% and increased heart rate variability by 16.9%. During sleep guanfacine increased HRV in the high frequency band [33]. Experiments using cardiac microdialysis showed that guanfacine can activate cardiac vagal nerves in anesthetized rabbit heart [34], which could increase HRV in the high frequency band.

Flow mediated dilation (FMD) measures the ability of the arteries to dilate. Guanfacine improved brachial artery FMD in contrast to therapy with other antihypertensives that generally do not improve FMD [35]. Guanfacine treatment increased brachial artery dilation during reactive hyperemia by 57%. Sympathetic stimulation and episodic hypoxia reduces FMD [36, 37]. Decreasing the sympathetic tone in OSA subjects with α-agonist drugs might improve FMD. We found differences in the effect of HCTZ and guanfacine on nocturnal dipping, central BP, HRV, FMD and BRS. These effects have generally not been seen in beta blocker clinical trials. HCTZ increases sympathetic tone and guanfacine decreases sympathetic tone. These opposing effects may have provided greater contrast than seen in studies where one drug is compared to placebo. Since these findings are novel, they deserve repeat study.

## Conclusions

Subjects with OSA had increased nighttime natriuresis and had elevated NE and E levels not only at night but also throughout the day. Treatment with the sympatholytic guanfacine lowered 24 h BP and BP dipping in OSA more than treatment with a diuretic. In addition, guanfacine improved HRV, baroreflex sensititivity and FMD while the diuretic had no effect. Treatment with calcium channel blockers, diuretics and vasodilators elicit further reflex activation of sympathetic nerves and should make BP even more sensitive to sympatholytics. Treatment resistant hypertension is often associated with OSA [2] and sympatholytics may assist BP control in these poorly responsive patients. The logic of using sympatholytic therapy to treat a condition such as OSA with increased sympathetic nerve activity [3] is straightforward and might apply to other conditions that increase sympathetic nerve activity.

### Electronic supplementary material

Below is the link to the electronic supplementary material.

Supplementary Material 1

## Data Availability

All data generated or analyzed during this study are included in this published article [and its supplementary information files].

## Abbreviations

OSA: Obstructive sleep apnea
BP: Blood pressure
SBP: Systolic blood pressure
DBP: Diastolic blood pressure
ANP: Atrial natriuretic peptide
BNP: B type natriuretic peptide
UCSD: University of California San Diego
HCTZ: Hydrochlorothiazide
AHA: American Heart Association
ABP: Ambulatory blood pressure
ABPM: 24 h ambulatory blood pressure monitoring
AHI: Apnea-hypopnea index
ESS: Epworth Sleepiness Scale
cfPWV: Carotid-femoral pulse wave velocity
PWA: Pulse wave analysis
ECG: Electrocardiogram
cBRS: Cardiovagal baroreflex sensitivity
HRV: Heart rate variability
FMD: Flow mediated dilation
ANOVA: Analysis of variance
NE: Norepinephrine
E: Epinephrine
BPV: Blood pressure variability
SD: Standard deviation
aAIx @ HR75: Augmentation index normalized at 75 beats per minute
ED%: Fractional ejection duration
RMSSD: Root mean square of successive RR interval differences
wSDf 24-h: diastolic blood pressure variability as weighted SD with fixed duration of the daytime (14 h) and nighttime (6 h) periods
wSDv 24-h: diastolic blood pressure variability as weighted SD with variable duration of sleep and wake periods
D: Brachial artery diameter
Db: Baseline diameter of brachial artery
D_max_: Maximal post occlusion brachial artery diameter
ΔD: Post occlusion brachial artery diameter change
V_avg_: Average brachial artery blood velocity during each cardiac cycle
AUC: Area under the curve
nFMD: Normalized flow mediated dilation
HF: High frequency band
LF: Low frequency band
