# Supplementary material for: Sympathetic overactivity and nocturnal diuresis in obstructive sleep apnea alter the response to hypertension therapy
Source: Clin Hypertens. 2024 Jun 1;30:14. doi: 10.1186/s40885-024-00272-x (PMC11143623; doi:10.1186/s40885-024-00272-x)
Supplement: Supplementary file 1 — Supplementary Material 1 [file 40885_2024_272_MOESM1_ESM.docx]

**Additional file 1**

**Table S1** Characteristics of study subjects in Study 1.

| Variables (mean±SD) | Healthy Controls | OSA |
| --- | --- | --- |
| N | 20 | 41 |
| Age (years)^*^ | 48±8.7 | 46.5±8.9 |
| Weight (kg) | 83.4±15.1 | 92.8±20.2 |
| Height (cm) | 172±8.5 | 177±9 |
| Body mass index (kg/m^2^) ^*^ | 27.9±3.8 | 29.5±5.5 |
| Systolic blood pressure (mmHg) ^**^ | 121±13 | 130±18 |
| Diastolic blood pressure (mmHg) | 76±9 | 78±9 |
| Heart rate (bpm) | 68±11 | 76±12 |
| Apnea/hypopnea index^**^ | 5.4±2.8 | 39±30 |
| Race |  |  |
| White | 11 | 37 |
| Black or African American | 9 | 2 |
| Asian |  | 1 |
| Other |  | 1 |
| Gender |  |  |
| Male | 15 | 37 |
| Female | 5 | 4 |

^*^*P* < 0.05; ^**^*P* < 0.01

**Table S2.** Plasma NE and E concentrations in Study 1.

| Plasma NE & E  (pg/mL±SEM) | Healthy Controls  N=20 | OSA  N=41 | *P* |
| --- | --- | --- | --- |
| NE Overall 24-h | 359±13 | 449±11 | <0.001 |
| NE Wake period | 393±18 | 474±15 | <0.001 |
| NE Sleep period | 312±19 | 416±16 | <0.001 |
| E Overall 24-h | 31±12 | 44±1 | <0.001 |
| E Wake period | 33±2 | 47±2 | <0.001 |
| E Sleep period | 29±2 | 41±2 | <0.001 |

**
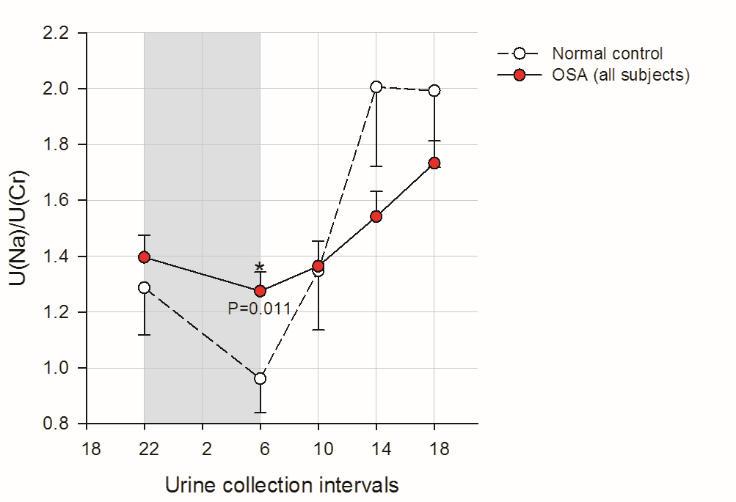
**

**Figure S1.** 24-h urine sodium to creatinine [U(Na)/U(Cr)] ratio in Study 1. OSA subjects have a greater [U(Na)/U(Cr)] during the nighttime collection; *P* > 0.05. Differences were NS at other collection points.

**Table S3.** Characteristics of OSA subjects in study 2.

| Variables | Mean ± SD |
| --- | --- |
| N | 23 |
| Age (years) | 52±11 |
| Weight (kg) | 105±24 |
| Height (cm) | 173±13 |
| Body mass index (kg/m^2^) | 35.2±8.6 |
| Systolic blood pressure (mmHg) | 139±12 |
| Diastolic blood pressure (mmHg) | 83±8 |
| Heart rate (bpm) | 72±10 |
| Apnea/hypopnea index | 26.2±11.5 |
| Race |  |
| White | 22 |
| Black or African American | 1 |
| Gender |  |
| Male | 20 |
| Female | 3 |

**Table S4.** Mean of average ambulatory BP, pulse pressures and heart rates during the overall 24h period, wake period and sleep period in Study 2.

|  | Treatment |  |  |  | *P*-values |  |  |
| --- | --- | --- | --- | --- | --- | --- | --- |
| Variable | Placebo | Guanfacine | HCTZ |  | Guanfacine  vs.  Placebo | HCTZ  vs. Placebo | Guanfacine  vs.  HCTZ |
| Overall 24h |  |  |  |  |  |  |  |
| SBP (mmHg) | 142±0.95 | 132±1.34 | 136±0.96 |  | P < 0.05 | P < 0.05 | P < 0.05 |
| DBP (mmHg) | 86±1.08 | 79±0.96 | 83±0.97 |  | P < 0.05* | P = 0.08 | P < 0.05 |
| MBP (mmHg) | 104±1.06 | 97±1.06 | 101±0.96 |  | P < 0.05 | P < 0.05 | P < 0.05 |
| PP (mmHg) | 57±0.43 | 53±0.5 | 54±0.44 |  | P < 0.05 | P < 0.05 | NS |
| HR (bpm) | 81±1.6 | 76±1.05 | 82±1.29 |  | P < 0.05 | NS | P < 0.05 |
| Wake period |  |  |  |  |  |  |  |
| SBP (mmHg) | 146±1.06 | 136±1.39 | 139±0.74 |  | P < 0.05* | P < 0.05 | P < 0.05 |
| DBP (mmHg) | 91±1.5 | 83±0.96 | 85±0.67 |  | P < 0.05* | P < 0.05 | P < 0.05 |
| MBP (mmHg) | 109±1.08 | 101±1.06 | 86±0.6 |  | P < 0.05* | P < 0.05 | P < 0.05 |
| PP (mmHg) | 57±0.58 | 54±0.7 | 54±0.44 |  | P < 0.05 | P < 0.05 | NS |
| HR (bpm) | 87±1.04 | 80±0.5 | 85±1.22 |  | P < 0.05 | NS | P < 0.05 |
| Sleep period |  |  |  |  |  |  |  |
| SBP (mmHg) | 136±0.69 | 126±1.8 | 131±1.08 |  | P < 0.05 | P < 0.05 | P < 0.05 |
| DBP (mmHg) | 82±1.32 | 74±1.33 | 80±1.65 |  | P < 0.05 | P < 0.05 | P < 0.05 |
| MBP (mmHg) | 98±0.53 | 92±1.39 | 97±1.7 |  | P < 0.05 | P < 0.05 | P = 0.06 |
| PP (mmHg) | 56±0.87 | 52±0.5 | 53±0.85 |  | P < 0.05 | P < 0.05 | P = 0.07 |
| HR (bpm) | 72±0.84 | 69±1.21 | 75±0.93 |  | NS | P < 0.05 | P < 0.05 |

*: Friedman RM ANOVA on Ranks.

**Table S5.** Ambulatory blood pressure variability determined by SD.

|  | Treatment |  |  |  | *P*-value |  |  |
| --- | --- | --- | --- | --- | --- | --- | --- |
| Variable | Placebo | Guanfacine | HCTZ |  | Guanfacine  vs.  Placebo | HCTZ  vs.  Placebo | Guanfacine  vs.  HCTZ |
| Overall 24h SD |  |  |  |  |  |  |  |
| SBP (mmHg) | 13.65±0.8 | 14.51±0.83 | 13.61±0.65 |  | 0.303 | 0.952 | 0.194 |
| DBP (mmHg) | 10.84±0.63 | 11.33±0.56 | 11.43±0.56 |  | 0.448 | 0.370 | 0.886 |
| MBP (mmHg) | 11.58±0.7 | 12.32±0.56 | 11.96±0.56 |  | 0.225 | 0.612 | 0.546 |
| Wake period SD |  |  |  |  |  |  |  |
| SBP (mmHg) | 11.92±0.74 | 12.98±0.88 | 12.46±0.56 |  | 0.236 | 0.484 | 0.556 |
| DBP (mmHg) | 9.59±0.62 | 10.07±0.67 | 10.19±0.54 |  | 0.440 | 0.702* | 0.869 |
| MBP (mmHg) | 10.24±0.68 | 11.02±0.64 | 10.81±0.52 |  | 0.071 | 0.651* | 0.424* |
| Sleep period SD |  |  |  |  |  |  |  |
| SBP (mmHg) | 12.25±0.77 | 13.24±0.87 | 12.09±0.91 |  | 0.286 | 0.885* | 0.325 |
| DBP (mmHg) | 9.3±0.58 | 10.11±0.47 | 9.58±0.75 |  | 0.274 | 0.768 | 0.489 |
| MBP (mmHg) | 9.99±0.68 | 11.17±0.73 | 10.28±0.82 |  | 0.135 | 0.754 | 0.361 |

SD: standard deviation, *: Friedman RM ANOVA on Ranks.

In contrast to conventional 24-h SD of BP, which is significantly affected by nocturnal BP dipping, the weighted 24-h SD (wSD) eliminates this unwanted effect and was proposed as the better index of the overall 24-h BP variability [25]. We calculated the overall 24-h SD of BP as a weighted SD (Table S6) by using two fixed time windows [25]:

$$\mathrm{wSD}_{f}=\frac{\left( daytime SD X 14 \right)+\left( nighttime SD X 6 \right)}{20}$$

We also calculated wSD_v_ by using two variable time windows. In this approach the duration of the sleep window is variable; it starts with sleep start and ends with sleep end (Table S6). The BP values recorded 2 hours prior to sleep start and 2 hours after awakening were excluded. There were no significant differences in weighted SBP and DBP variabilities between HCTZ and guanfacine. However, guanfacine significantly increased weighted 24-h DBP variability compared to placebo (Table S6).

**Table S6.** Overall 24-h ambulatory blood pressure variability determined by weighted SD.

|  |  |  |  | *P*-value |  |  |
| --- | --- | --- | --- | --- | --- | --- |
| Variable | Placebo | Guanfacine | HCTZ | Guanfacine  vs.  Placebo | HCTZ  vs.  Placebo | HCTZ  vs. Guanfacine |
| Overall 24h wSDf |  |  |  |  |  |  |
| SBP (mmHg) | 8.99±0.66 | 10.26±0.7 | 10.19±0.7 | 0.155 | 0.193 | 0.940 |
| DBP (mmHg) | 7.17±0.53 | 8.03±0.47 | 8.18±0.7 | 0.024* | 0.213 | 0.845 |
| MBP (mmHg) | 7.65±0.61 | 8.79±0.58 | 8.74±0.78 | 0.089 | 0.273 | 0.954 |
| Overall 24h wSDv |  |  |  |  |  |  |
| SBP (mmHg) | 9.16±0.59 | 10.26±0.68 | 9.45±0.54 | 0.217 | 0.721 | 0.294 |
| DBP (mmHg) | 7.03±0.54 | 7.95±0.48 | 7.65±0.6 | 0.025* | 0.702* | 0.672 |
| MBP (mmHg) | 7.62±0.65 | 8.84±0.59 | 8.23±0.69 | 0.082 | 0.614* | 0.465 |

wSD_f_ : weighted standard deviation with fixed time window, wSD_v_: weighted standard deviation with variable time window, *: Friedman RM ANOVA on Ranks.

**Table S7.** Nocturnal blood pressure dipping.

|  |  |  |  |  | *P*-value |  |  |
| --- | --- | --- | --- | --- | --- | --- | --- |
| Variable | Placebo | Guanfacine | HCTZ |  | Guanfacine  vs.  Placebo | HCTZ  vs.  Placebo | HCTZ  vs. Guanfacine |
| SBP(%) | 6.6±1.8 | 9.1±1.2 | 6.6±1.4 |  | 0.103 | 0.978 | 0.029* |
| DBP(%) | 11.5±1.5 | 13.3±1.5 | 12.4±1.6 |  | 0.278 | 0.588 | 1* |

SBP(%): percent of nocturnal systolic BP dip, DBP(%): percent of nocturnal diastolic BP dip,

*: Friedman RM ANOVA on Ranks.

**Table S8.** Central pulse wave analysis (PWA).

|  | Treatment |  |  |  | *P*-value |  |  |
| --- | --- | --- | --- | --- | --- | --- | --- |
| Variable | Placebo | Guanfacine | HCTZ |  | Guanfacine vs.  Placebo | HCTZ vs. Placebo | Guanfacine vs.  HCTZ |
| bSBP (mmHg) | 144±5 | 122±3 | 134±3 |  | < 0.05 | < 0.05 | < 0.05 |
| bDBP (mmHg) | 85±3 | 71±2 | 83±2 |  | < 0.05 | NS | < 0.05 |
| bMBP (mmHg) | 104±3 | 88±2 | 100±2 |  | < 0.05 | NS | < 0.05 |
| bPP (mmHg) | 59±3 | 50±2 | 51±3 |  | < 0.05* | NS* | NS* |
| HR (bpm) | 74±3 | 67±3 | 73±2 |  | < 0.05 | NS | < 0.05 |
| aSBP (mmHg) | 129±5 | 109±3 | 120±3 |  | < 0.05 | < 0.05 | < 0.05 |
| aDBP (mmHg) | 86±3 | 72±2 | 84±2 |  | < 0.05 | NS | < 0.05 |
| aMBP (mmHg) | 104±3 | 88±2 | 100±2 |  | < 0.05 | NS | < 0.05 |
| aPP (mmHg) | 43±3 | 37±2 | 36±2 |  | < 0.05 | < 0.05 | NS* |
| aAP (mmHg) | 9±1 | 8±1 | 6±1 |  | NS | NS | NS |
| aAIx@HR75 (%) | 19.0±1.3 | 14.7±1.5 | 15.2±2 |  | <0.05 | <0.05 | NS |
| ED (%) | 35±1 | 32±1 | 34±1 |  | < 0.05 | NS | < 0.05 |
| SEVR (%) | 161±5.6 | 180±6.9 | 170±6.2 |  | < 0.05 | NS | NS |
| Tr (s) | 141±2 | 145±2 | 143±2 |  | NS | NS | NS |

b: brachial, a: aortic, AP: augmented pressure, PP: pulse pressure, aAIx@HR75: augmentation index normalized at heart rate 75 bpm, ED%: fractional ejection duration, SEVR: subendocardial viability ratio, Tr: pulse wave travel time, *Friedman RM ANOVA on Ranks.

**Table S9.** Carotid-femoral Pulse Wave Velocity (cfPWV).

|  | Treatment |  |  |  | *P*-value |  |  |
| --- | --- | --- | --- | --- | --- | --- | --- |
| Variable | Placebo | Guanfacine | HCTZ |  | Guanfacine  vs.  Placebo | HCTZ  vs. Placebo | Guanfacine  vs.  HCTZ |
| cfPWV (m/s) | 7.6±0.4 | 7±0.3 | 7.3±0.4 |  | NS | NS | NS |
| bSBP (mmHg) | 133±4 | 119±3 | 129±4 |  | < 0.05 | NS | < 0.05 |
| bDBP (mmHg) | 76±2 | 69±2 | 74±2 |  | < 0.05 | NS | NS |
| Distance (mm) | 424±9 | 425±9 | 418±9 |  | NS | NS | NS |

cfPWV: carotid-femoral pulse wave velocity, b: brachial.

**Table S10.** Heart rate variability (HRV).

|  | Treatment |  |  |  | *P*-value |  |  |
| --- | --- | --- | --- | --- | --- | --- | --- |
| Variable | Placebo | Guanfacine | HCTZ |  | Guanfacine  vs.  Placebo | HCTZ  vs.  Placebo | Guanfacine  vs.  HCTZ |
| HR (bpm) | 68 ± 2 | 63±2 | 68±3 |  | <0.05 | NS | <0.05 |
| RMSSD (ms)* | 36.7 | 42.9 | 35.1 |  | <0.05 | NS | NS |
| LF power (ms^2^)† | 358 | 365 | 242 |  | NS | NS | NS |
| HF power (ms^2^)† | 216 | 332 | 139 |  | NS | NS | <0.05 |
| Total power (ms^2^)† | 1430 | 1793 | 999 |  | NS | NS | NS |
| LF (nu) | 59.86±5.34 | 50.99±5.42 | 61.59±3.76 |  | NS | NS | NS |
| HF (nu) | 40.14±5.34 | 49.01±5.42 | 38.41±3.76 |  | NS | NS | NS |
| LF/HF ratio* | 1.73 | 0.87 | 1.51 |  | NS | NS | NS |

HR: heart rate, RMSSD: root mean square of successive RR interval differences, LF: low frequency,

HF: high frequency, nu: normalized units, *: median, †: geometric mean.

**Table S11.** Spontaneous cardiovagal baroreflex sensitivity (cBRS).

|  | Treatment |  |  |  | *P*-value |  |  |
| --- | --- | --- | --- | --- | --- | --- | --- |
| Variable | Placebo | Guanfacine | HCTZ |  | Guanfacine vs.  Placebo | HCTZ  vs.  Placebo | Guanfacine  vs.  HCTZ |
| cBRS(ms/mmHg) | 7.70 ± 0.89 | 11.37 ± 1.56 | 8.50 ± 1.63 |  | <0.05 | NS | NS |
| SBP (mmHg) | 142 ± 4.7 | 130 ± 2.2 | 137 ± 3.5 |  | <0.05 | NS | NS |
| DBP (mmHg) | 77 ± 2.1 | 71 ± 2.2 | 73 ± 1.7 |  | <0.05 | NS | NS |
| IBI (ms) | 906 ± 32 | 980 ± 40 | 883 ± 32 |  | <0.05 | NS | <0.05 |
| TPR (dyn.s/cm^5^) | 1060 ± 98 | 990 ± 102 | 895 ± 80 |  | NS | NS | NS |
| SV (mL) | 96.42 ± 6.69 | 101.20 ± 7.71 | 105.27 ± 8.18 |  | NS | NS | NS |

cBRS, cardiovagal baroreflex sensitivity; SBP, mean beat to beat systolic BP; DBP, mean beat to beat diastolic BP; IBI, mean interbeat interval; TPR, mean beat to beat total peripheral resistance; SV, mean beat to beat stroke volume;
